# Supplementary material for: Oxidative Glucose Metabolism Promotes Senescence in Vascular Endothelial Cells
Source: Cells. 2022 Jul 16;11(14):2213. doi: 10.3390/cells11142213 (PMC9322806; doi:10.3390/cells11142213)
Supplement: Supplementary file 1 [file cells-11-02213-s001.zip › cells-1768512-supplementary.pdf]

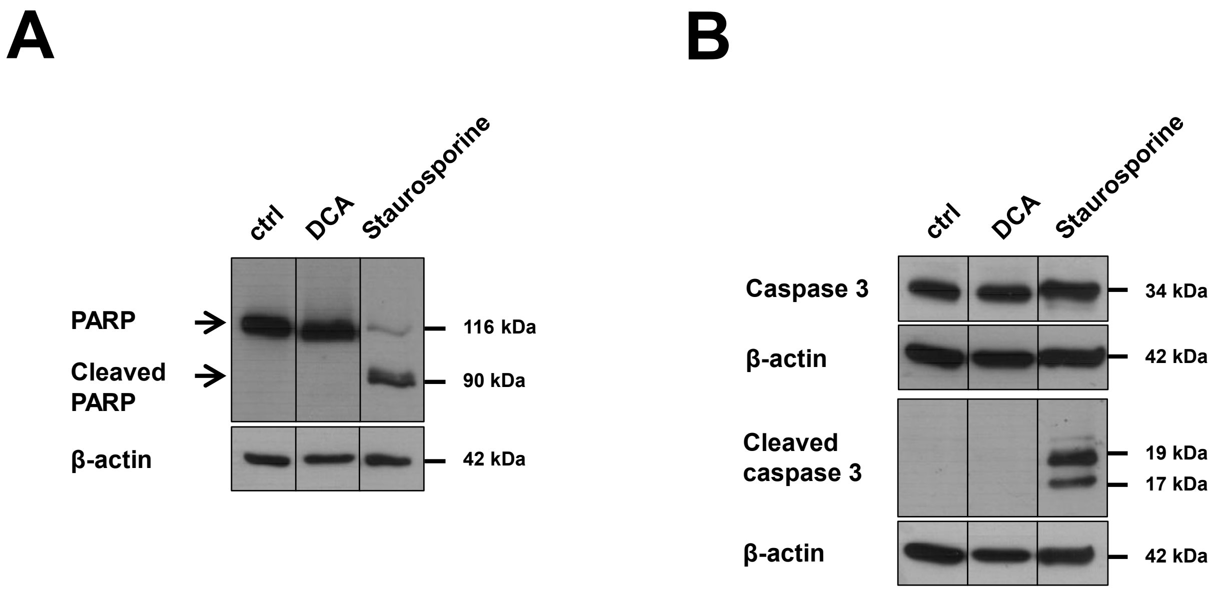

Supplementary figure S1:
Dichloroacetate treated endothelial cells show no signs of apoptosis. Primary non-senescent HUVEC were left untreated (control, ctrl) or treated with 20 mM of dichloroacetate (DCA) for 24 h. Treatment with 5 µM staurosporine for 2 h was used as a positive control. (A) Representative western blot of PARP and Cleaved PARP (n=4). (B) Representative western blot of Caspase 3 and Cleaved caspase 3 (n=4). *PARP= poly ADP ribose polymerase.*

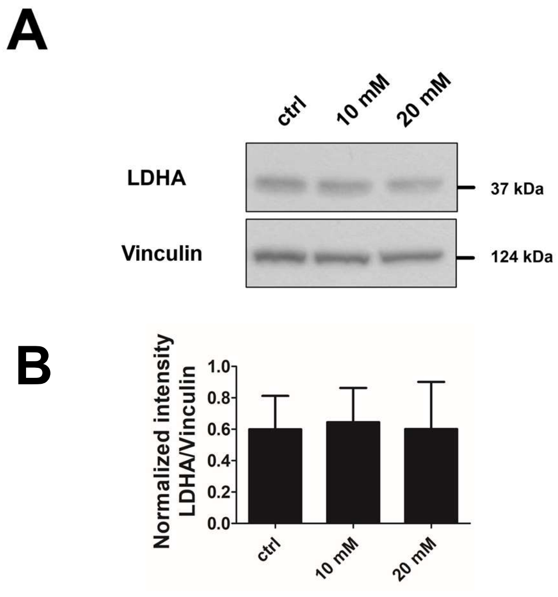

Supplementary figure S2:
Dichloroacetate does not affect LDHA expression. Primary non-senescent HUVEC were left untreated (control, ctrl) or treated with 10 mM or 20 mM dichloroacetate (DCA) for 24 h. (A) Upper panel: Representative western blot of LDHA. (B) Lower panel: Densitometric quantification of LDHA after normalization to vinculin (n=4, repeated measure (RM) one-way ANOVA $p>0.05$).
